# Relations between Spatial Distribution, Social Affiliations and Dominance Hierarchy in a Semi-Free Mandrill Population

**DOI:** 10.3389/fpsyg.2016.00612

**Published:** 2016-05-03

**Authors:** Alexandre Naud, Eloise Chailleux, Yan Kestens, Céline Bret, Dominic Desjardins, Odile Petit, Barthélémy Ngoubangoye, Cédric Sueur

**Affiliations:** ^1^Centre National de la Recherche Scientifique, Département Ecologie, Physiologie, et EthologieStrasbourg, France; ^2^Département de Médecine Sociale et Préventive, École de Santé Publique, Université de MontréalMontréal, QC, Canada; ^3^Institut Pluridisciplinaire Hubert Curien, Université de StrasbourgStrasbourg, France; ^4^École Nationale Vétérinaire d'AlfortMaison-Alfort, France; ^5^Jr Research Group “Sexual Selection,” German Primate CenterGöttingen, Germany; ^6^Départements des Sciences Biologiques, Institut de Recherche en Biologie Végétale, Université de MontréalMontréal, QC, Canada; ^7^Centre International de Recherches Médicales de FrancevilleFranceville, Gabon

**Keywords:** mandrill, spatial distribution, feeding competition, dominance, affiliative relationships, social network

## Abstract

Although there exist advantages to group-living in comparison to a solitary lifestyle, costs and gains of group-living may be unequally distributed among group members. Predation risk, vigilance levels and food intake may be unevenly distributed across group spatial geometry and certain within-group spatial positions may be more or less advantageous depending on the spatial distribution of these factors. In species characterized with dominance hierarchy, high-ranking individuals are commonly observed in advantageous spatial position. However, in complex social systems, individuals can develop affiliative relationships that may balance the effect of dominance relationships in individual's spatial distribution. The objective of the present study is to investigate how the group spatial distribution of a semi-free ranging colony of Mandrills relates to its social organization. Using spatial observations in an area surrounding the feeding zone, we tested the three following hypothesis: (1) does dominance hierarchy explain being observed in proximity or far from a food patch? (2) Do affiliative associations also explain being observed in proximity or far from a food patch? (3) Do the differences in rank in the group hierarchy explain being co-observed in proximity of a food patch? Our results showed that high-ranking individuals were more observed in proximity of the feeding zone while low-ranking individuals were more observed at the boundaries of the observation area. Furthermore, we observed that affiliative relationships were also associated with individual spatial distributions and explain more of the total variance of the spatial distribution in comparison with dominance hierarchy. Finally, we found that individuals observed at a same moment in proximity of the feeding zone were more likely to be distant in the hierarchy while controlling for maternal kinship, age and sex similarity. This study brings some elements about how affiliative networks and dominance hierarchy are related to spatial positions in primates.

## Introduction

Group living is a common social pattern among primates (Alexander, 1974; Wrangham, 1987). Although there exist advantages of group-living in comparison to a solitary lifestyle (Krause and Ruxton, 2002), a growing body of evidence indicates that costs and benefits of group living may be unequally distributed and spatially determined (Viscido and Wethey, 2002; Quinn and Cresswell, 2006; Hirsch, 2007; Tkaczynski et al., 2014). Research suggests that predation risk, vigilance levels, and food intake may depend on an individual's position within a group spatial geometry. This implies that certain within-group spatial positions may be more or less advantageous than others (Janson, 1990; Krause, 1994; Motro et al., 1996; Hall and Fedigan, 1997; Hirsch, 2007) and that individuals may compete for certain spatial positions (Motro et al., 1996) or adopt particular spatial behaviors (De Vos and O'Riain, 2010) in order to maximize their fitness. Furthermore, in species characterized by a dominance hierarchy, high-ranking individuals are commonly observed in more advantageous spatial positions (i.e., a position that reduces costs and maximize the gains of group-living; van Noordwijk and van Schaik, 1987; Janson, 1990; Hall and Fedigan, 1997; Murray et al., 2007).

(Hamilton, 1971) selfish herd theory suggests that individuals mainly located at the edge of a group should experience higher risk of predation in comparison to their central counterparts. This “marginal effect” is well-supported by empirical evidence on different taxa, which demonstrates that predation risk (Krause, 1994; Stankowich, 2003) and vigilance levels (Petit and Bildstein, 1987; Janson, 1990; Burger et al., 2000) tend to be higher among peripheral individuals. Even when predators have relatively equal access to all group members—i.e., when predators move into a 3 dimensional space while prey move in a 2 dimensional space—they are more likely to attack peripheral animals (Romey et al., 2008). This means that in order to reduce one's risk of predation, individuals will compete for a central group position (Couzin and Krause, 2003) resulting in group aggregation (Hamilton, 1971). In addition, studies on inter-individual spacing have shown that groups tend to become more tightly spaced after an encounter with a predator (van Schaik and Mitrasetia, 1990; De Vos and O'Riain, 2010) or in high predation risk areas (Quinn and Cresswell, 2006; Kelley et al., 2011).

Food gain has also been found to be related to spatial position (Hirsch, 2007). When food is dispersed, spread, and thus not monopolized (scramble competition), foraging gains may increase for peripheral group members as spacing between them reduces feeding competition (Morrell and Romey, 2008). Conversely, when food patches are limited and defendable (i.e., contest competition; van Schaik and van Noordwijk, 1988), individuals may aggressively compete over food (Grant et al., 2002) resulting in a spatial distribution where high-ranking individuals are in the center, occupying food patches, while low-ranking individuals are distributed in peripheral positions (Hirsch, 2007). This spatial distribution, characterized by dominant and tolerated individuals in central positions having high food intake, is observed in different primate species (Robinson, 1981; Janson, 1990; Barton, 1993; Motro et al., 1996). When we consider group mobility, the most advantageous position in species following a producer-scrounger model (i.e., individuals found their own food—produce—or join the food discoveries of others—scrounger–) should be in the center-front during group foraging (Hirsch, 2007) which has been observed in white-faced capuchins (*Cebus capucinus*; Robinson, 1981; Hall and Fedigan, 1997).

Dominance hierarchy may not be the only social variable that explains within group spatial distributions among primate species that live in relatively stable groups (i.e., where relationships persist over months or even years). In complex social systems (Whitehead, 2008), individuals might develop affiliative relationships that shape social organization (Pasquaretta et al., 2014). These affiliations may therefore balance the effect of dominance relationships in individual's spatial distribution. Indeed, subordinate individuals tend to groom high ranked individuals (Schino, 2001; Nakamichi and Shizawa, 2003; Silk et al., 2003) presumably to develop alliances and tolerance in order to increase access to resources and to get potential allies in agonistic interaction (Seyfarth, 1977). This might change the ranks of these subordinate individuals or give them access to an advantageous spatial position without changing ranks. Additionally, Robinson (1981) has shown that an individual's spatial location is best predicted when affiliative relationships are considered during agonistic interactions. These strategies and social preferences make the emergent group social network more complex. At a population level, association preferences between multiple individuals may divide the community into subgroups where individuals in a subgroup interact more among themselves than with the rest of the community (Krause et al., 2007; Sueur et al., 2011a). Such community divisions, or clusterisations, potentially resulting in fission-fusion dynamics (Sueur et al., 2011c), have been observed in different primate species (Mandrills, *Mandrillus sphinx*: (Bret et al., 2013)*;* Rhesus macaques, *Macaca mulatta* and Japanese macaques, *Macaca fuscata*: (Sueur et al., 2011d); Howling Monkeys, *Alouatta palliata*: (Bezanson et al., 2008); Human, *Homo sapiens*: Newman, 2004) and other non-primate social species (Bottlenose dolphin, *Delphinidea Tursiops*: (Lusseau et al., 2006); Columbian ground squirrel, *Spermophilus columbianus*: Manno, 2008). Clusterisations may play a role in competition for a certain spatial location—low-ranking individuals in a subgroup with high-ranking individuals may access food patches more easily—or may help to decrease food competition by spreading individuals across different resources (Sueur et al., 2011b).

The present study investigates how the group spatial distribution of a semi-free ranging colony of Mandrills (*M. sphinx*) relates to its social organization. Mandrills are highly social primates found in large groups (i.e., hordes) comprised of several hundred individuals in a natural context (Rogers et al., 1996; Abernethy et al., 2002). Social organization of the Mandrill in a natural context is poorly understood (Setchell and Wickings, 2005). Abernethy et al. (2002) has described Mandrill groups as stable and possessing a social organization consisting of adult females and their dependent offspring. Less than 2% of the group is constituted of adult males, while other adults and sub-adult males are present only during the mating season. Only the alpha male is 100% permanently associated with the social group (Setchell and Dixson, 2001). Previous studies of a semi-free-ranging colony showed that male Mandrills exhibit a strong linear dominance hierarchy and that affiliative behavior is extremely rare among males (Setchell and Wickings, 2005; Setchell et al., 2006). On the other side, females seem to be organized in matrilines (Setchell, 1999) and kin related females fraternize together more than unrelated females (Bret et al., 2013). A more recent study using social network analyses has shown that semi-free mandrills are organized in subgroups of preferential relationships, which are not related to kinship, age, or dominance rank of group members (Bret et al., 2013). The absence of a correlation between kinship and subgroup organization in this study may be explained by group composition (i.e., some females were the only representatives of their matriline). However, it may suggest that affiliative relationships also shape the social organization of Mandrill living in semi-free conditions.

In our study, we aim to examine, in a semi-free mandrill population, how individual's spatial position during food competition is explained by group social organization. Previous studies have shown that dominance relationships may explain the spatial distribution of individuals according to food distribution pattern, with dominant individuals monopolizing resources. Our objective is to evaluate whether affiliative relationships also explain individual spatial distribution in this situation. Our research questions are: (1) does dominance hierarchy explain observed proximity or distance from a food patch? (2) Do affiliative associations also explain observed proximity or distance from a food patch? (3) Do rank differences in the group hierarchy explain being co-observed in proximity of a food patch?

Our expected findings are that high-ranking individuals will be observed in proximity of the food patch more often, while low-ranking individuals will remain distant from the food patch. We also expect that belonging to certain subgroups of affiliative relationships will also explain spatial observations within different distances of the food patch, while controlling for dominance ranking. This would suggest that affiliative relationships are another aspect of social organization that may explain access to advantageous spatial positions. Finally, we have two opposite hypotheses for our third research question. Individuals of similar rank in the hierarchy may form stronger bonds than individuals of distant rank, as usually individuals of neighboring ranks are more closely related (Cheney and Seyfarth, 1990) and are more tolerant of each other. In this case, individuals of a similar rank should be co-observed more often in close proximity of the food patch. In contrast, previous studies found that proximity (or distance) within the dominance hierarchy does not explain affiliation in semi-free Mandrills (Bret et al., 2013) and they seem to be characterized by a more relaxed dominance hierarchy in comparison to other primate populations (Bout and Thierry, 2005). Therefore, it is possible that distant individuals within the hierarchy may tolerate each other in proximity of the feeding area.

## Methods

### Ethical statement

Our methodological approach solely involved observations. Animals were not handled, and no invasive experiments were carried out on the mandrills. Animals were already accustomed to human presence in their enclosure. Our protocol followed the ethical guidelines of the CNRS (Centre National de Recherche Scientifique) and the recommendations of the Gabonese government. This study was conducted with the approval of the International Medical Research Center (CIRMF) scientific committee in Gabon via a research agreement (nu045/2011/CNRS). All occurrences of injuries or illness in the observed animals were reported to veterinary staff at the CIRMF primatological center.

### Study group and environment

The study was comprised of 39 mandrills from a group of 75 individuals born in captivity and living in a large, naturally rainforested enclosure (6.5 ha), at the CIRMF in Franceville, Gabon. Mandrills were free to forage in the enclosure and were supplemented by a provision of homemade soya-cake and local seasonal fruits twice a day. Water was available *ad libitum*. Juveniles (<5 years old) were excluded from the study population because they spent all their time with their mothers and because of the instability of their relationships with other group members (Sueur et al., 2011d). Remaining individuals were aged between 5 and 26 years (mean = 10.42; SD = 4.09) and comprised of 18 females and 21 males. Dates of birth and matrilineage were recorded for all individuals. Kinship was computed from matrilineage by recording motherhood. All subjects were identified using morphological differences and/or ear tags.

### Spatial observations and data recording

**Figure 1:**
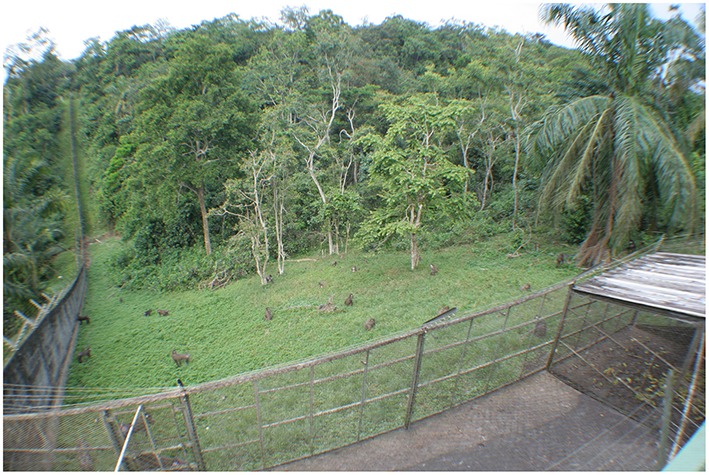
**Photograph of the observation area from the tower where observations were realized**. Credit Chailleux E.

**Figure 2:**
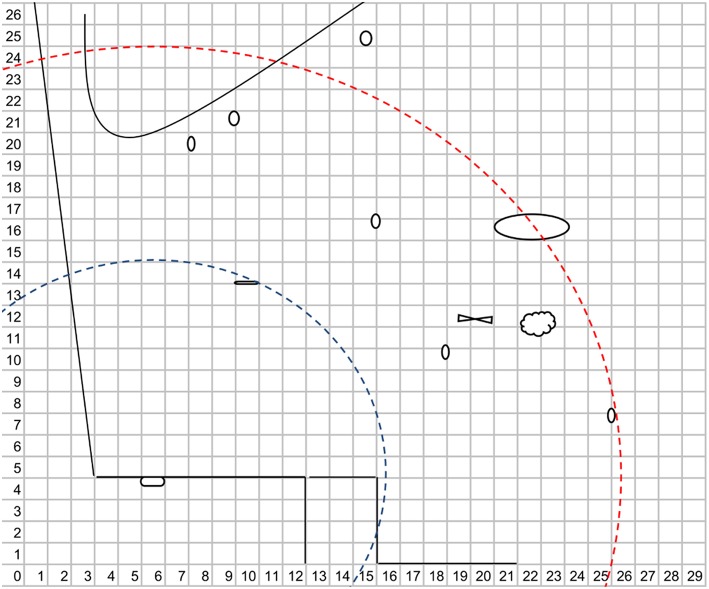
**Schema used to record individuals' spatial position**. The scale of this schema is 1 m^2^. Circles, ellipses, and polygons represent trees and bushes where the mandrill could get covered. The line on the upper side represents the beginning of the forest area. The feeding zone is contained to X = 12–15 and Y = 0–4 and the door is represented by the polygon in X = 5–6 and Y = 3–4. The two circular buffers are characterized by the 10 m (blue) and the 20 m (red) areas used to calculate frequencies of observation.

Data was collected from April to June 2012. One of the researchers (E.C.) observed the group 6 h per day (09:00–12:00 and 15:00–18:00) from a tower located behind the feeding zone. Observations were recorded in front of the feeding zone in a 30 × 30 m area covered with grass and small trees allowing good visibility (see Figure 1). At the beginning of each observation period (i.e., a.m. and p.m.), a food supplement was placed in a closed section, visible but not accessible to the mandrills during the whole observation period. At the end of each observation period (i.e., after 12:00 and 18:00), the door giving access to the food supplement was opened. This situation resulted in an artificial food patch, an advantageous spatial position for which the Mandrills could compete for. We used instantaneous scan sampling (Altmann, 1974) with a 15 min sampling frequency (total of 26 scans per day) to record individual spatial positions within the observation area. Individuals observed during a scan were positioned on a map representing the 30 × 30 m area in front of the feeding zone door using 1 m spaced vertical and horizontal grid lines (Figure 2). A total of 631 scans were completed, representing 157.75 h of observations. All individuals were not observed at each scan (mean frequency of scans = 145.2; SD = 73.35).

**Figure 3:**
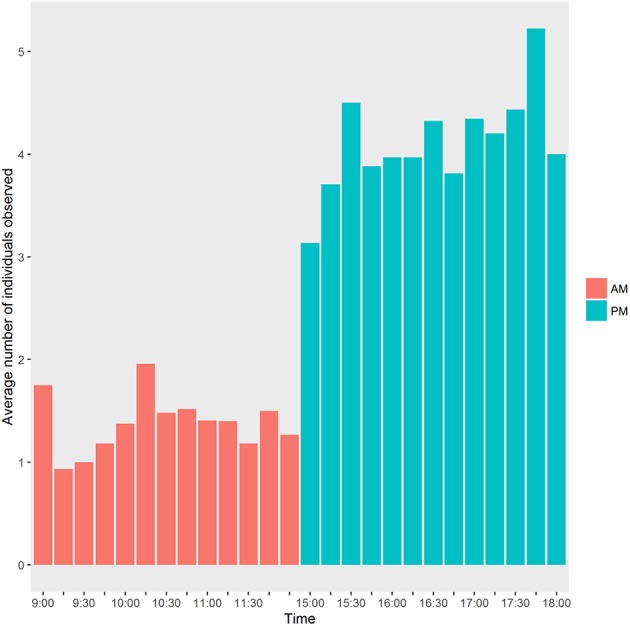
**Histogram of the average number of individuals observed during each period**. 12:00 is considered in the morning period (am) in this figure. The bar plot was realized with the ggplot2 package (Wickham, 2009).

When calculating the mean number of observed individuals by observation period (e.g., 9:00, 9:15), we noticed that more individuals were observed during the afternoon (i.e., 15:00–18:00) compared to the morning (i.e., 09:00–12:00; see Figure 3). In practice, the food was not always made available by the CIRMF at 12:00 but systematically at 18:00. Therefore, mandrills had possibly learned this pattern and food competition was probably more important during the afternoon. Consequently, we used only observations from the afternoon period to calculate spatial positions of individuals within different distances of the feeding zone (see Section Hierarchal Dominance).

### Spatial distribution

First, we calculate the Euclidian distance (Legendre and Legendre, 2012) between the center of the feeding zone door and the Cartesian coordinates of all observations (Figure 2). We used the door as the centroid of the food patch because this location was the most coveted since it is the only access to the feeding zone. Then, the frequency of observations within 10 m and over 20 m distances of the food patch were computed from the Euclidian distances. We did not use observations between 10 and 20 m because we wanted to contrast spatial observations in proximity and distant from the feeding zone door. We used a 10 m scale in order to get sufficient observations for statistical analysis. For observations within 10 m of the feeding door, we calculated the relative frequencies to adjust for the number of scans during which individuals were observed. For observations over 20 m of the feeding door, individuals that were out of sight (i.e., in the forest area) were included in the measure. Two variables were created: (1) the relative frequency of observation within 10 m of the feeding door (F10M) and (2) the frequency of observations over 20 m of the feeding door (F20M).

### Hierarchal dominance

Agonistic interactions were recorded *ad libitum* during both observation periods (i.e., a.m. and p.m.). According to previous studies (Setchell, 1999), a list of 13 behaviors was chosen and described in a catalog. Actor, receivers, behavior, date, and time were recorded for each aggressive event. When interactions included a series of behaviors, only the last behavior (causing the submission of the other mandrill) was recorded. Also, only unidirectional aggression with a clear issue was used to calculate the hierarchy. Since dominance hierarchy is only based on dyadic interactions, interactions between more than two individuals were discarded. Linearity of the hierarchy was measured with de Vries' *h'* index (de Vries, 1995). Individual dominance indices were calculated with de Vries' modification of David's score (MDS) (David, 1987; de Vries et al., 2006). These measures were calculated with SOCPROG 2.4 (Whitehead, 2009).

### Affiliative relationships and co-occurrence within 5 m of the feeding zone

To measure the social network of affiliative relationship (i.e., affiliative network), we used spatial proximity (i.e., association measures) between individuals as a proxy of social preferences in our group (Whitehead, 2008). Two individuals (i.e., a dyad) were considered to be in association when they were seen within a distance of 1 m from each other during a scan. We assumed that a distance close to the average body length of adult mandrills represented a situation where touch interactions (e.g., grooming) may take place. The average body length of adult mandrills is 80 cm for males and 60 cm for females (Wickings and Dixson, 1992). We were, however, limited to 1 m because it was the smallest distance measured from the previous spatial observations (the data were already collected when the analyses were planned). For each pair of individuals, we calculated a half-weight association index (HWI) with the number of associations. Since we did not observe the whole group at each scan, all individuals were not observed at the same total frequency. HWI allow to control for the non-observation of all group members at each scan (Whitehead, 2008). Then, we tested if individuals associated in a non-random way by permuting associations within each scan (*H0* = no preferred or avoided relationship for any dyad; Whitehead et al., 2005). We used the coefficient of variation of association indices as the test statistic for significance level. To measure if our population could be usefully divided into subgroups, we used the modularity test of Newman (Newman, 2004, 2006) which measures the difference between the proportion of the total associations in the subgroups and the summed associations of the whole group. Eigenvalues were used to determine the level of certainty through which individuals were assigned to subgroups. These three measures were computed with SOCPROG 2.6 (Whitehead, 2009).

Finally, we created a second association matrix where every individual observed within 5 m of the feeding zone door in a same scan were considered associated (i.e., co-occurrence network). We used HWI to control for non-observation of group members. We used a smaller radius than for spatial proximity to the feeding zone door (5 m instead of 10 m) because we assumed that competition would be stronger in a limited space and therefore co-occurrence would indicate tolerance among individuals.

### Statistical analyses

To evaluate homoscedasticity assumptions, we used Spread-Location plots of standardized residuals against fitted values, Bartlett test, and the Breusch-Pagan test (Greene, 2003; Scherrer, 2007). We used square root transformations on the relative frequency within 10 m (SRF10M) in order to obtain homoscedasticity and reduce the skewness of the distribution. We had two missing values for the variable age. We replaced those values by the means of their respective age group (e.g., sub adults' mean ages = 6.8). We had one missing data for MDS and removed the individual from further analysis. All significance levels were obtained through permutations because our observations did not represent a sample from a larger statistical population with a known distribution and permutations allow parametric statistical methods to be used when distributional assumptions are not satisfied (Legendre and Legendre, 2012). We used 10,000 permutations in each analysis.

First, we aimed to test how hierarchy is structured by age and sex by testing bivariate relationships between hierarchy, sex, and age. Relationship between dominance rank (MDS) and sex was tested using a student test (one-tailed). Relationships between dominance rank and age were tested on the full sample and stratified by sex using Pearson correlation tests (one-tailed). We used one-tailed tests because our hypothesis was that males were more dominant than females and there is a positive correlation between age and dominance ranking. Second, we tested the relationship between the explanatory variables (1) age, (2) sex, (3) dominance rank, and (4) matrilineage (i.e., kinship) and affiliative associations. Age, dominance rank, and sex variables were first transformed into three distance matrices. Distances for age and dominance ranking were calculated with Euclidian distance and sex was calculated with a binary coefficient (same sex = 1; different sex = 0). We then used a multiple regression quadratic assignment procedure (MRQAP) test (Whitehead, 2008) with the “double-semi-partialing” technique of Dekker et al. (2007). This analysis aimed to better understand how social relationships are distributed according to animal characteristics.

To test our first research question—does dominance hierarchy explain observed proximity or distance from a food patch?—we used Pearson correlations (one-tailed) between dominance rank and frequency of observations within distances of the feeding door (SRF10M and F20M). To test our second research question—do affiliative associations also explain observed proximity or distance from a food patch?—we used partial regression to test the relationships between SRF10M or F20M as outcomes, and dominance rank and subgroups (found with modularity method as described above) as explanatory variables. Finally, to test our third hypothesis—do rank differences in the group hierarchy explain being co-observed in proximity of a food patch?—we tested the correlation between the co-occurrence network and the dominance distance matrix while controlling for kinship, age, and sex with the MRQAP test using the “double-semi-partialing” technique. Correlation were run between the co-occurrence network and the affiliative network (recalculated without observation within 5 m of the feeding zone) with a Mantel test to see if the co-occurrence within 5 m of the feeding door was related to the observed associations in the whole area.

All statistics were performed in R 3.1.2. (CRAN, 2014) and SOCPROG 2.6. (Whitehead, 2009). We used Bartlett-test (stats library) and ncvTest (car package) functions for Bartlett and Breusch-Pagan tests, corPerm3 and t.perm (available from Pierre Legendre website) functions for bivariate relationships, mantel.test (ape package) function for mantel test, varpart and rda (vegan package) functions for partial regressions (Paradis et al., 2004; Fox and Weisberg, 2010; Legendre, 2015; Oksanen et al., 2016). MRQAP tests were performed in SOCPROG 2.6.

## Results

### Dominance hierarchy

**Figure 4:**
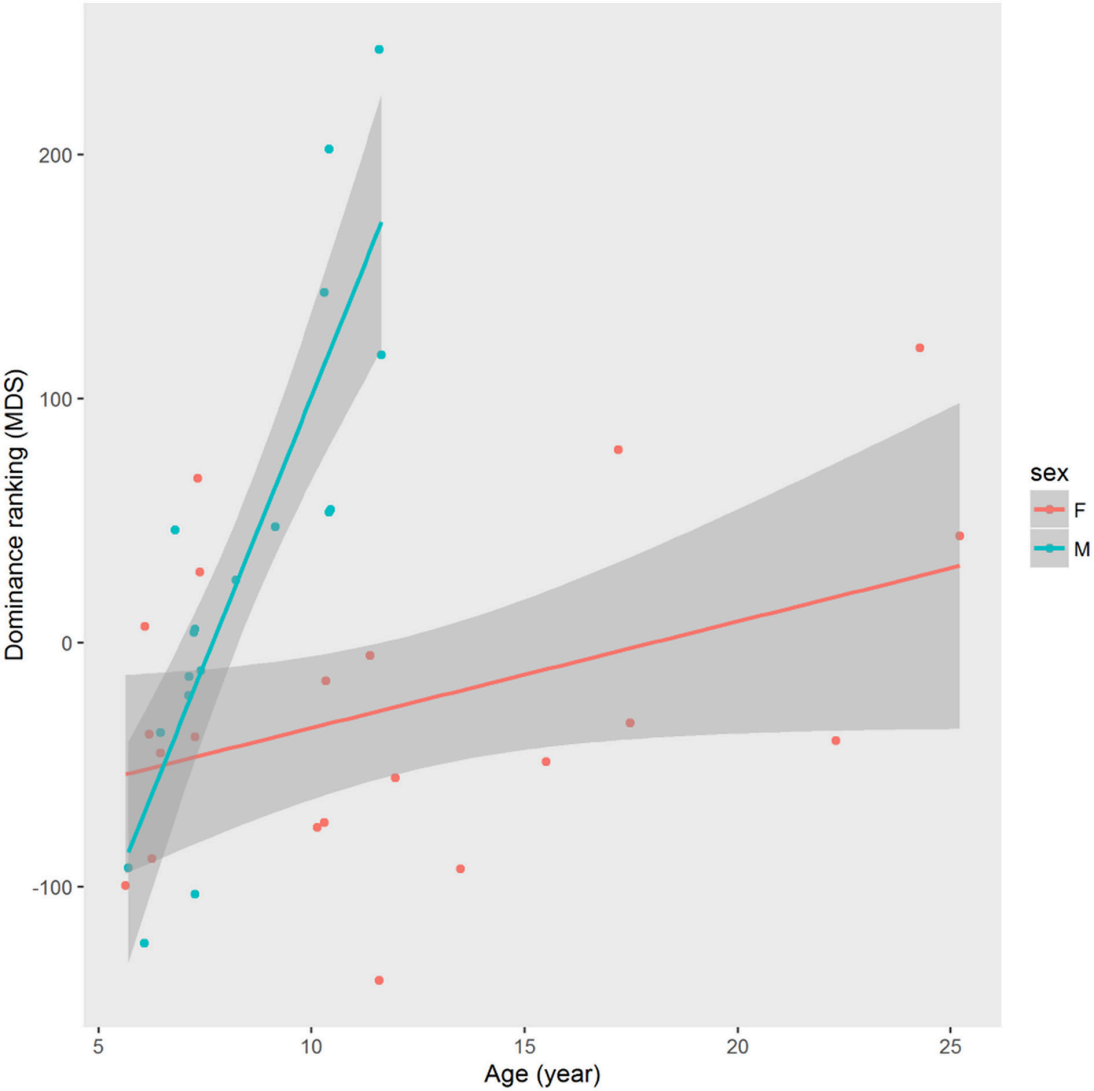
**Linear regression between dominance rank (MDS) and age (years) in semi-free ranging group of mandrills (***Mandrillus sphynx***) stratified by sex**. The line represents the curve estimated by the linear model and the gray shape represents the standard error. The scatter plot was realized with the ggplot2 package (Wickham, 2009).

Linearity of the dominance hierarchy was significant [p(perm) < 0.0001] but not perfectly consistent [De Vries h' = 0.253]. Dominance index (MDS) was correlated to sex [p(perm) = 0.02053] where males had greater dominance indices in comparison to females. The correlation between dominance index and age was not significant for the whole group [*r* = 0.24924; p(perm) = 0.07069] but significant when stratified by sex. Stratified linear correlation showed that this relationship is stronger for males [*r* = 0.88461; p(perm) < 0.00001] than for females [*r* = 0.41382; p(perm) = 0.03548; Figure 4].

### Affiliative relationships

**Figure 5:**
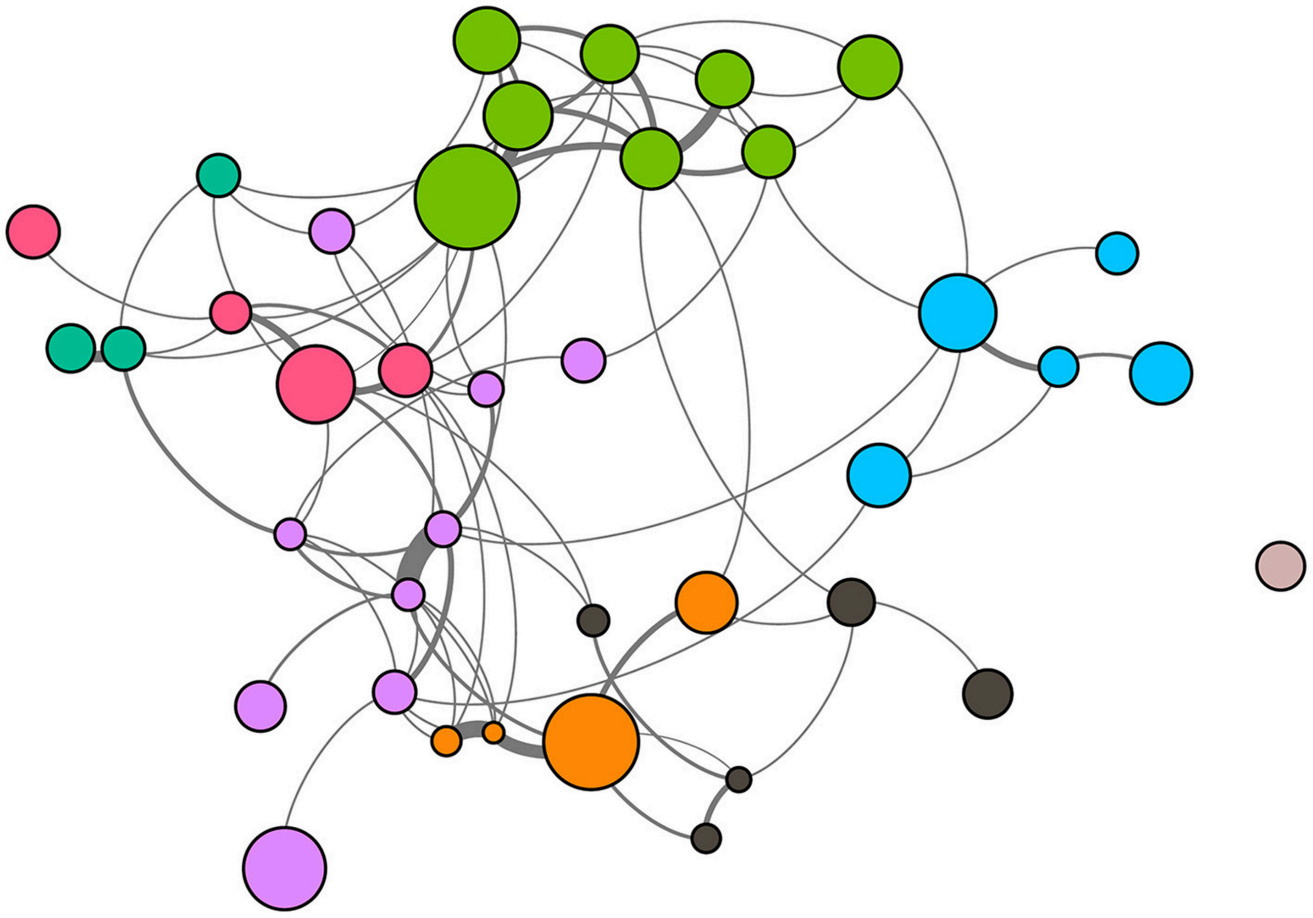
**One meter proximity network of a semi-free ranging group of mandrills (***Mandrillus sphynx***)**. This network was generated from the matrix of associations. Node size represents variation in hierarchical dominance rank (MDS). Node color shades characterize subgroup membership and edge thickness represents the strength of the connection between two nodes; the thicker the edge, the stronger the association. Individuals are positioned in 2D according to their social relationships using the Force Atlas 2 spatialization option in Gephi 0.9 (Bastian et al., 2009).

The estimated affiliative associations (Figure 5) were found to be non-random [p(perm) < 0.001]. Affiliative associations were not correlated with age differences [partial *r* = 0.0525; p(perm) = 0.1119] or with MDS differences [partial *r* = 0.0359; p(perm) = 0.1919], but were correlated with sex [partial *r* = 0.0858; p(perm) = 0.0099] and matrilineage [partial *r* = 0.3081; p(perm) < 0.0001] where individuals of the same sex and kin associate more often. Newman modularity tests gave us eight subgroups composed of 3–9 individuals. Modularity for this test was 0.590, and a modularity score > 0.3 indicates a useful subdivision of the group (Whitehead, 2008; Sueur et al., 2011b).

### Spatial positions according to dominance hierarchy and affiliative network (H1 and H2)

Proximity to the feeding zone was correlated with dominance hierarchy: SRF10M was positively correlated with MDS [one-tailed Pearson correlation; *r* = 0.36385; p(perm) = 0.01190] and F20M was negatively correlated with MDS [one-tailed Pearson correlation; *r* = −0.45755; p(perm) = 0.00155].

In the multivariate linear model with SRF10M as an outcome (Table 1), the fraction of variance explained by MDS [semipartial *r*^2^ = 0.089658; p(perm) = 0.015] and subgroups [semipartial *r*^2^ = 0.450367 p(perm) = 0.002] are both significant [adjusted *R*^2^ = 0.48853; p(perm) = 0.002]. In the multivariate linear model with F20M as an outcome, the fraction of variance explained by MDS [semipartial *r*^2^ = 0.15267; p(perm) = 0.003] and the fraction of variance explained by subgroups [semiparial *r*^2^ = 0.28086; p(perm) = 0.005] are both significant [adjusted *R*^2^ = 0.37510; p(perm) = 0.001; Table 1]. These results indicate that both MDS and subgroups predict being observed in SR10M and F20M when controlling for the other predictor, but that subgroups explain more of the total variance in both cases.

**Table 1:** **Partial regressions between each spatial observation variables (SRF10M, F20M) as outcomes and dominance hierarchy (MDS) and subgroups as explanatory variables**.

	**Df**	**Semipartial *r*^2^**	**Adjusted *R*^2^**	**p(perm)**
**SRF10M**				
MDS	1	0.089658		0.015
Subgroups	6	0.450367		0.002
MDS + Subgroup	7		0.48853	0.002
**F20M**				
MDS	1	0.15267		0.003
Subgroups	6	0.28086		0.005
MDS + Subgroup	7		0.3751	0.001

### Co-occurrence network and dominance hierarchy (H3)

The co-occurrence network (individuals co-observed within 5 m of the feeding zone) was correlated to a second affiliative network calculated without associations within 5 m of the feeding zone [Mantel test; *r* = 0.2169; p(perm) < 0.0001]. When using binary descriptors (1 = affiliation; 0 = no affiliation), 76% of the affiliative associations found within 5 m of the feeding zone were observed in the rest of the area (i.e., second affiliative network). We found a positive correlation between the co-occurrence network and the dominance distance matrix [partial *r* = 0.2046; p(perm) = 0.0007] while controlling for kinship [partial *r* = 0.0751; p(perm) = 0.0449], age distance [partial *r* = −0.0061, p(perm) = 0.4489], and sex similarity [partial *r* = 0.0928; p(perm) = 0.0117], which indicates that individuals that are co-observed more often around the feeding zone door have greater differences in their respective dominance status while controlling for the other descriptors.

## Discussion

In this study, we aimed to understand how the spatial position of individuals in a feeding context was influenced by social organization.

We first found that dominance hierarchy was correlated with sex, where males were more dominant than females within the group. We also found linear correlations between dominance and age within male and female individuals. These results are coherent with previous studies on the same study population and from a captive population (Holt, 1980; Setchell et al., 2006). Studies on mandrills remain rare and these results are important to understand the social organization of this species. The distribution of relationships within our affiliative network was correlated with sex and kinship but not to age and dominance. In comparison with a previous study on a different Mandrill group living in the same semi-free context (CIRMF colonies), the correlations with age and dominance were found to be consistent, while correlations with sex and kinship were found to be inconsistent (Bret et al., 2013). A possible explanation for this difference comes from the composition of our study population. Few females were related in the previous study, whereas more females were from the same matriline in our study population. Our ability to compare this population to other semi-free populations is limited, since most of our knowledge on Mandrill behavior comes from the CIRMF colonies.

High-ranking individuals were more often observed in proximity of the feeding-zone area, while low-ranking individuals were more observed at the boundaries of the observation area. In our case, the food patch was limited in space and was only available for a fixed time. This encourages contest competition resulting in a spatial distribution where high-ranking individuals control the food patch and low-ranking individuals are in peripheral positions. This behavior has been observed in different primate species (Long-Tailed Macaques, *Macaca fascicularis*: van Schaik and van Noordwijk, 1988; Wild chimpanzees, *Pan troglodytes*: Murray et al., 2007; White-faced capuchins, *Cebus capuccinus*: Hall and Fedigan, 1997; Brown capuchin *Cebus apella*: Janson, 1990) and other taxa (Convict cichlids, *Archocentrus nigrofasciatum*: Grant et al., 2002). Furthermore, frequencies of the presence of observations, either in proximity or far from the feeding zone, were also explained by the affiliative network. Division of the affiliative network into subgroups of preferential associations explained ~45 and 28% of the total variation of observations within 10 m (SRF10M) and over 20 m (F20M) of the feeding zone, respectively. In comparison, dominance explained ~9 and 15% of the total variation of SRF10M and F20M, respectively [these percentages are the rounded semipartial *r*^2^ calculated in Section Spatial Positions According to Dominance Hierarchy and Affiliative Network (H1 and H2)]. These results do not only indicate that affiliative relationships are associated with individual spatial distributions, but that the subgroup associations explain more of the total variance in both cases. Therefore, spatial distribution in a feeding context seems to be more strongly associated with the affiliation than dominance hierarchy within the social organization. In this way, affiliative relationships may allow individuals to be tolerated by high-ranking individuals at the feeding zone and the resulting affiliative advantage for low-ranking individuals may explain the observed competition to groom dominant individuals (Sade, 1972; Chapais et al., 1995; Schino, 2001).

The analysis of the co-occurrence network showed that individuals observed within 5 m of the feeding zone at the same moment were more likely to be distant within the hierarchy, while controlling for maternal kinship, age differences, and sex similarity. Furthermore, the co-occurrence network was correlated with the affiliative network, which indicates that associations in the feeding zone area were consistent with associations observed in the whole area. A possible explanation for these results is that low-ranking individuals use their preferential associations with high-ranking non-kin individuals in order to gain access to the feeding-zone area. This would be consistent with results from another study on the wedge-capped capuchin monkeys (*Cebus nigrivittatus*), which found that food patches were controlled by the most dominant individuals, their siblings, and tolerated individuals (Robinson, 1981).

Dominance may be an important factor for accessing food when one is at the top of the hierarchy, but for mid and lower ranked individuals, affiliation through alliances with high-ranked individuals seems to be a more effective way of reaching food than relying strictly on one's own dominance status. A possible explanation for these results is the existence of a biological market between associated individuals of different dominance ranking (Barrett et al., 1999). Among wild tufted capuchin monkeys (*Cebus abella*) and wild Japanese macaques (*M. fuscata*), during feeding, high-ranking individuals are more likely to tolerate low-ranking individuals that groom them the most, after controlling for kinship (Ventura et al., 2006; Tiddi et al., 2011). Moreover, high-ranking individuals tend to be groomed more than their low-ranking counterparts, a phenomenon that is observed in adult females of different primate species (Schino, 2001). However, other studies on *C. abella* and *M. fuscata* found no correlation between dominance distances and grooming behaviors (Nakamichi and Shizawa, 2003; Schino et al., 2009). Tolerance is a currency that primates may exchange against affiliative behaviors (Janson, 1985), and therefore, low-ranking individuals may tend to groom high-ranking individuals so as to improve their fitness by gaining access to resources that are monopolizable (Fruteau et al., 2009). Furthermore, market exchange between allogrooming and agonistic support has also been observed among different primate species (Schino, 2007).

This study fulfilled its objective of better understanding the spatial position of mandrill group members under a feeding context but met some limitations. Whilst the study of captive populations might allow us to gain a better understanding of the social factors affecting behavior, our first limitation was that the phenomenon observed in a semi-free context may not be representative of the natural context of behavior. An example of this situation is found in the group clusterization: subgroup number three that consisted of five low-ranking adult males with five of the seven lowest positions in the male hierarchical ranking. These individuals would probably have left the population in a natural context since only dominant individuals remain in the population outside the mating season (Abernethy et al., 2002). In this semi-free context, they associated themselves with other mandrills and remained in the periphery of the group, mimicking males' migration observed in the wild (Abernethy et al., 2002). A second limitation was that we had no information on associations and tolerance when the feeding zone door was open. Thus, it is possible that high-ranking individuals became intolerant with low-ranking individuals when food was accessible. Another way of measuring food accessibility would have been by co-feeding in the feeding zone. Thirdly, we had no measures of grooming interactions and therefore, the association network may not be fully representative of the real occurrences of affiliative interactions. However, body contact and close proximity networks were correlated in the study of another group in the CIRMF, validating the use of close proximity (within 1 m) as a relevant variable to represent social relationships (Bret, personal comm.). Fourthly, the spatial distribution (Section Spatial Distribution) and social association (Section Affiliative Relationships and Co-Occurrence within 5 m of the Feeding Zone) measures were both derived from the same dataset (i.e., Cartesian coordinates within the 30 × 30 m area; Section Spatial Observations and Data Recording). This could have created dependence between our variables and thus may influence our results. For observations within 10 m of the food patch, Cartesian coordinates of dyadic associations during the afternoon period and within 10 m of the food patch were comprised in both measures (SF20M and affiliative relationship). These dyadic associations represented 12.7% of all observed dyadic associations. We performed sensitivity analysis with subgroups of preferential associations (Section Affiliative Relationships and Co-Occurrence within 5 m of the Feeding Zone) recalculated without these dyadic associations and found that relationships between spatial distribution, dominance index and subgroups [Section Spatial Positions According to Dominance Hierarchy and Affiliative Network (H1 and H2)] were consistent with our previous findings. We also found our results to be consistent with those of a sensitivity analysis performed with the observations that were over 20 m away from the food patch. Finally, even if we restricted our spatial distribution measures to the afternoon period, it is impossible to know whether mandrills maintained interest in an inaccessible food source for 3 h. Thus, not all these measures of spatial distribution may have been taken in a feeding competition context.

This study gives us a better understanding of how affiliative networks and dominance hierarchy are related to the spatial positions of primates. These results were obtained by combining social network analysis with spatial analysis. A next step would be to better understand the temporal dimension of this process. This would result in determining how aspects of social organization co-influence animal behavior and explain within group spatial distribution.

## Author contributions

AN is the main author of this article. He developed the hypothesis, conduct the analysis, and wrote the article. EC collected the data and assisted AN in the elaboration of the hypotheses, the analysis and the writing of this paper. DD and CB contributed to the analysis and the work drafting. YK and CS supervised the analysis and revised the work critically. OP and BN made possible the acquisition of the data used in this article. All authors agreed to be accountable for all aspects of the work in ensuring that questions related to the accuracy or integrity of any part of the work are appropriately investigated and resolved.

### Conflict of interest statement

The authors declare that the research was conducted in the absence of any commercial or financial relationships that could be construed as a potential conflict of interest.
